# 2MDR, a Microcomputer-Controlled Visual Stimulation Device for Psychotherapy-Like Treatments of Mice

**DOI:** 10.1523/ENEURO.0394-22.2023

**Published:** 2023-06-02

**Authors:** Isa Jauch, Jan Kamm, Luca Benn, Lukas Rettig, Hans-Christoph Friederich, Jonas Tesarz, Thomas Kuner, Sebastian Wieland

**Affiliations:** 1 Department of Functional Neuroanatomy, Institute for Anatomy and Cell Biology, Heidelberg University, 69120 Heidelberg, Germany; 2 Department of General Internal and Psychosomatic Medicine, Heidelberg University and Heidelberg University Hospital, 69115 Heidelberg, Germany

**Keywords:** bilateral stimuli, brain stimulation, EMDR, fear conditioning, PTSD, visual stimulation

## Abstract

Post-traumatic stress disorder and other mental disorders can be treated by an established psychotherapy called Eye Movement Desensitization and Reprocessing (EMDR). In EMDR, patients are confronted with traumatic memories while they are stimulated with alternating bilateral stimuli (ABS). How ABS affects the brain and whether ABS could be adapted to different patients or mental disorders is unknown. Interestingly, ABS reduced conditioned fear in mice. Yet, an approach to systematically test complex visual stimuli and compare respective differences in emotional processing based on semiautomated/automated behavioral analysis is lacking. We developed 2MDR (MultiModal Visual Stimulation to Desensitize Rodents), a novel, open-source, low-cost, customizable device that can be integrated in and transistor–transistor logic (TTL) controlled by commercial rodent behavioral setups. 2MDR allows the design and precise steering of multimodal visual stimuli in the head direction of freely moving mice. Optimized videography allows semiautomatic analysis of rodent behavior during visual stimulation. Detailed building, integration, and treatment instructions along with open-source software provide easy access for inexperienced users. Using 2MDR, we confirmed that EMDR-like ABS persistently improves fear extinction in mice and showed for the first time that ABS-mediated anxiolytic effects strongly depend on physical stimulus properties such as ABS brightness. 2MDR not only enables researchers to interfere with mouse behavior in an EMDR-like setting, but also demonstrates that visual stimuli can be used as a noninvasive brain stimulation to differentially alter emotional processing in mice.

## Visual Abstract

**Figure:**
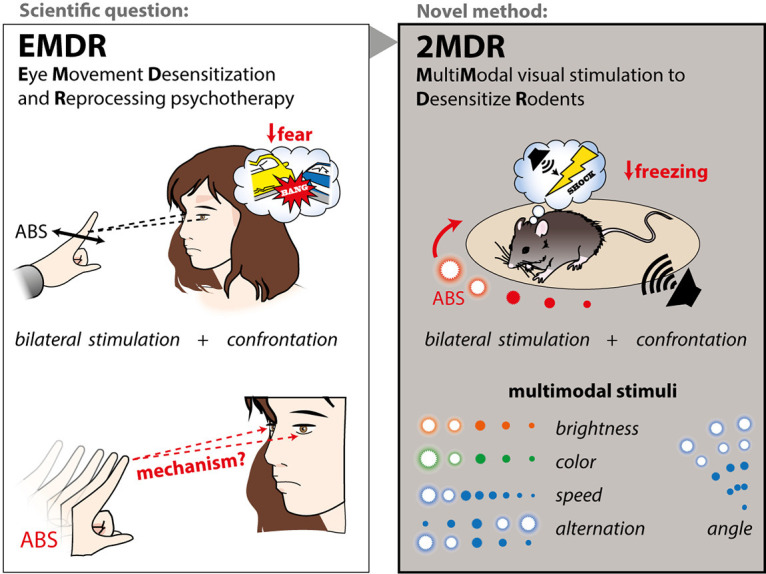

## Significance Statement

Alternating bilateral stimuli (ABS) reduce fear in post-traumatic stress disorder patients and in mice. The mechanism of how classic ABS—typically used in Eye Movement Desensitization and Reprocessing (EMDR)—reduces fear is enigmatic. We provide detailed resources to build a cost-effective, computer-controlled device called 2MDR to perform and semiautomatically analyze EMDR-like treatments in freely moving mice and to test behavioral effects of multiple ABS variants. Using the 2MDR device, this study confirmed that classic ABS strongly and persistently improves the extinction of conditioned fear in mice, an effect that depended on the brightness of ABS. This novel method may ultimately contribute to a deeper translational and neurobiological understanding of how visual stimuli affect emotional processing in mice.

## Introduction

Alternating bilateral stimuli (ABSs) are the key component of an established psychotherapy against post-traumatic stress disorder (PTSD) called Eye Movement Desensitization and Reprocessing (EMDR; Shapiro, 1989; Van Ommeren, 2013). In EMDR, a patient is recalling a traumatic memory while being exposed to classic ABSs. Classic ABSs are defined in that the patient shifts the gaze to pursue a visual stimulus such as a light beam or the therapist’s hand that horizontally oscillates at a stable frequency of ∼1 Hz. Combining exposure with classic ABSs improves the extinction of traumatic memories and reduces hyperarousal, and vividness of memories and intrusions (Calancie et al., 2018; Rousseau et al., 2019). EMDR has been adapted to many mental disorders (e.g., chronic pain, and depressive or anxiety disorders) through disorder-specific exposure protocols (Feske and Goldstein, 1997; Tesarz et al., 2013, 2014; Fereidouni et al., 2019), but not through significant adaptations of the classic ABS paradigm. Despite its clinical evidence, it remains unresolved how ABSs alter emotional processing in patients (Calancie et al., 2018). A better mechanistic understanding would provide insights into whether ABS variants could further improve EMDR treatments of specific disorders or individuals.

Human research suggests that EMDR differs from conventional exposure therapies as ABSs directly affect the processing of traumatic memories (Lee and Cuijpers, 2013). Recent animal studies revealed a deeper neurobiological understanding of ABS-mediated effects (Wurtz et al., 2016; Baek et al., 2019). In these studies, researchers simultaneously presented both the ABS and the fear-associated cue to persistently reduce conditioned fear and revealed an ABS-mediated activation of a novel circuit in the superior colliculus (SC), which is known for multisensory integration and saccade control, that suppressed cue-related fear engrams in amygdala.

An animal ABS stimulation model allows the investigation of interactions between ABSs and the brain not only on the level of brain circuits but also on a procedural level of respective ABS properties. With more options in terms of standardization and feasibility compared with human studies (Boukezzi et al., 2017), a suitable animal ABS stimulation model could elucidate which ABS properties causally mediate the anxiolytic effects in mice and which ABS variant optimally reduces fear. To investigate the influence of physical ABS properties on mice, researchers need a system that is capable of creating a plethora of stimulus qualities and of enabling standardized and EMDR-like experiments in mice. Unfortunately, there are no commercial systems available to perform such rodent experiments.

We successfully built a customizable, low-cost device allowing the experimenter to design and center various visual stimuli in the head direction of freely moving mice. We share detailed instructions on hardware and software to ease the setup process for inexperienced researchers. Furthermore, we validated our system and confirmed that classic ABSs persistently improve fear extinction (FE) in mice and showed for the first time that these anxiolytic effects depend on specific physical properties of ABSs, such as ABS brightness. This new method has the potential to substantially improve the study of EMDR and of noninvasive visual brain stimulation in rodents.

## Materials and Methods

### System overview and integration

**Figure 1.**
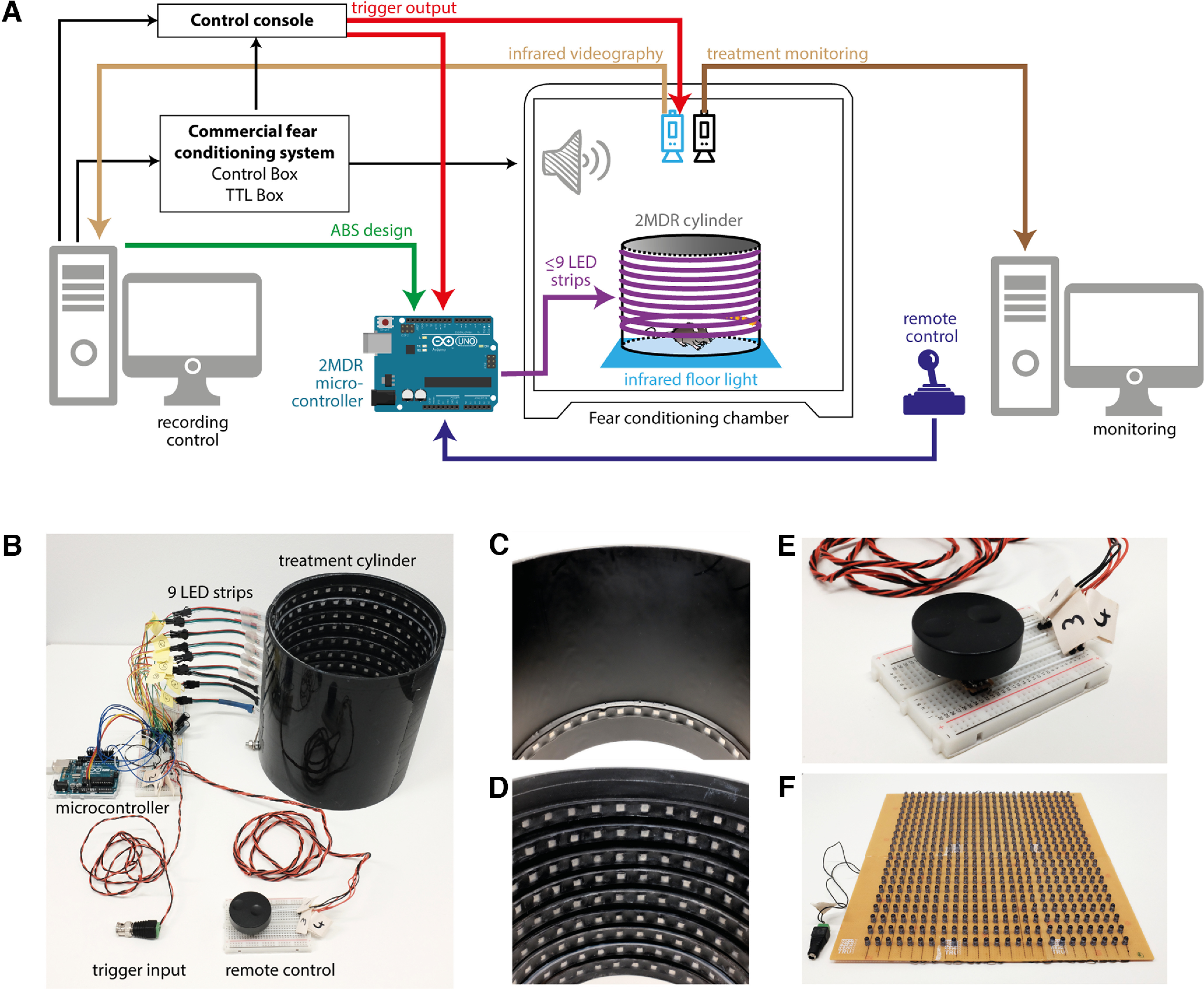
Overview and integration of the 2MDR system in a fear conditioning setup. ***A***, Schematic overview of how to integrate 2MDR in a commercially available fear conditioning system. ***B–E***, Photographs of the 2MDR system. ***B***, Overview showing all electronic components including the remote control, trigger input, and treatment cylinder. ***C***, ***D***, Two example configurations of the treatment cylinder with a single row (***C***) and 9 rows (***D***) of LED strips. ***E***, Closeup image of the remote control. ***F***, Low construction of the infrared floor illumination panel made of a 25 × 20 LED array (Extended Data Fig. 1-1, more details of construction, Movie 1, details of ABS patterns, Movie 2, rotary control of ABSs).

**Figure 2.**
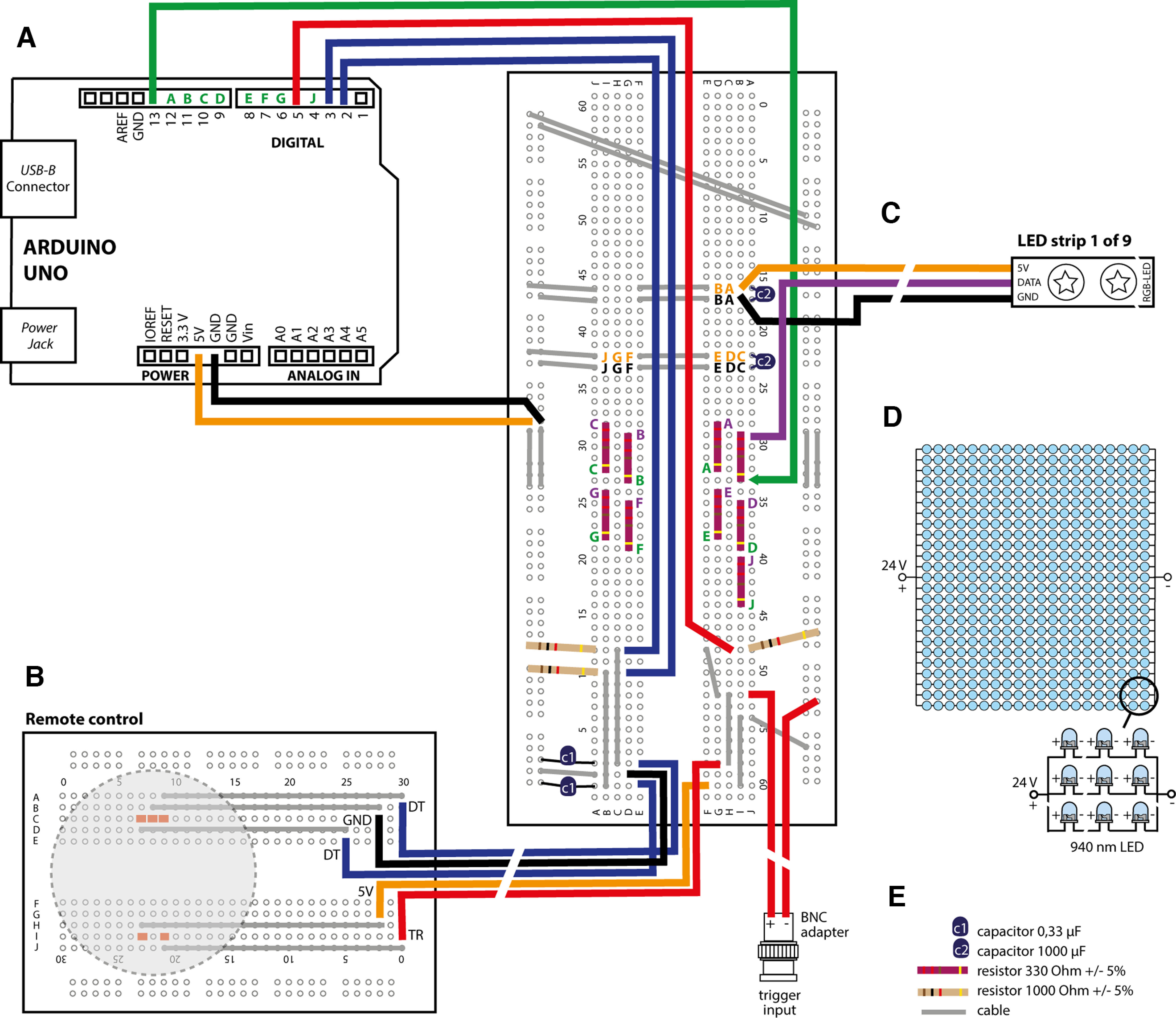
2MDR hardware components. ***A***, A schematic showing the hardware part of 2MDR based on an microcontroller (Uno, Arduino). ***B***, GPIO pins 2 and 3 receive rotation data (dark blue) of remote control. ***C***, Arduino GPIO pins 4 and 6–13 convey data of ABS design and movement (green) to control LED strips (Extended Data Figure 2-1, details of ABS user interface). GPIO pin 5 receives trigger information (red) from external trigger input or manual trigger input from remote control. H, We displayed the connection principle of one of the nine LED strips (LED strip), whereas the connections of the other eight LED strips are color coded for clarity. A–J, Each LED strip needs four cable connections [green pins, connection between board and the Uno microcontroller board (Arduino); violet pins, connection between board and LED strip data input; orange pins, 5 V supply; black pin, ground circuit]. ***D***, A schematic of our infrared floor light showing a parallel circuit of 25 rows of 20 LEDs (wavelength, 940 nm; Extended Data Fig. 2-2, Movie 3, more details of infrared videography. ***E***, Visual figure legend.

Our MultiModal Visual Stimulation to Desensitize Rodents (2MDR) device consists of an Arduino-based microcontroller, a treatment cylinder, a remote control, and infrared videography based on an infrared floor illumination (Fig. 1*A*,*B*). The main electronic component of the 2MDR apparatus (Table 1) is a microcontroller board (Uno, Arduino; https://www.arduino.cc; Fig. 2*A*, Extended Data Fig. 1-1*A*), which is programmed with a custom integrated development environment (Arduino)-based script that encodes ABS characteristics. We provide four baseline scripts to generate either horizontally, vertically, or two different patterns of oppositely oscillating visual stimuli to investigate anxiolytic effects of different types of stimulation (Movie 1). All four scripts work in an analogous fashion and are commented for users without coding expertise (Extended Data Fig. 2-1). The following parameters can easily be defined in our scripts: stimulus width (number of LEDs used), alternation frequency (in hertz), stimulus color and brightness, stimulus duration after trigger and row pattern. The digital output signals of the microcontroller board (Uno, Arduino) communicate with up to nine LED strips (model APA104, SparkFun; Figs. 1*D*, 2*C*) via general purpose input/output (GPIO) pins (Extended Data Fig. 1-1*B*). Each LED strip contains 36 LEDs under a waterproof silicone shield (Extended Data Fig. 1-1*D*,*E*). These LED strips are attached to the walls of a black vinyl or acrylic cylinder, in which the visual stimuli can be presented to the mice. The number and alignment of individual LED strips can be flexibly chosen for every setup to perform experiments with different visual stimulus patterns. We recommend covering the inside of the cylinder with a nonreflective, resistant coating [e.g., ultramat twi-component paint (Crop 2K, SprayMax; RAL 9005, Ultrimax)] or alternatives with transparent ultramat paint (Aerodecor transparent matt, Modulor) to carefully prevent light scattering during ABS application (Fig. 1*C*).

**Table 1:** List of components necessary to build one 2MDR hardware system

Item	Supplier/product number	Quantity	Price (€)
Arduino Uno - R3	Mouser Electronics/PN 782-A000066	1	27.60
LED RGB strip	Mouser Electronics/PN 474-COM-15205	6	26.00
Jumper wires M/F (10 pieces)	Mouser Electronics/PN 474-PRT-09140	4	3.95
Breadboard self-adhesive	Mouser Electronics/PN 474-PRT-12002	2	4.95
USB-A to USB-B cable	Mouser Electronics/PN 562–3021062-06	1	2.75
Power supply plug	Mouser Electronics/PN 418-TRE15050E11G03VI	1	11.23
BNC connector	Mouser Electronics/PN 992-BNC-F-TERM-S	1	6.04
Capacitor 1000 μF	Mouser Electronics/PN 474-COM-08982	2	0.39
Capacitor 0.33 μF	Mouser Electronics/PN 647-UFG1HR33MDM1TD	2	0.31
Resistor 330 Ω	Mouser Electronics/PN 71-CCF07330RJKE36	9	0.18
Resistor 1000 Ω	Mouser Electronics/PN 71-CPF11K0000FKE14	3	2.05
Jumper wires M/M 6in (10 pieces)	Mouser Electronics/PN 474-PRT-11709	1	5.95
Jumper wires M/M 7in (30 pieces)	Mouser Electronics/PN 474-PRT-11026	1	2.25
Jumper wire kit (140 pieces)	Mouser Electronics/PN 474-PRT-00124	1	6.57
Rotary encoder	Mouser Electronics/PN 706-61C11-01–08-01	1	35.28
Knob for rotary encoder	Mouser Electronics/PN 706-11K5013-KCNG	1	1.76
Superglue CA 100	Modulor online shop/PN 0154792	1	15.50
Ultramat black spray paint 2K Spraymax	Driessen International/CROP PN 2K-ZWART-SPUITBUS	1	18.86
Total price			324.66

For the cylinder built, we used either black acrylic glass or polyvinylchloride tubes (wall thickness, 4 mm). We used tubes with a diameter of 20 cm and a height of 20 cm with a slit-like cut or drilled hole access for the LED strips (Extended Data Fig. 1-1).

10.1523/ENEURO.0394-22.2023.f1-1 Figure 1-1 2MDR hardware. Photographs showing hardware details of 2MDR. ***A***, Details of microcontroller und breadboard. ***B***, Breadboard shown in high resolution. ***C***, Remote control–rotary encoder connections. ***D***, ***E***, Slit-like (***D***) and hole-like (***E***) entry of LED strip to cylinder. The hole-like entry was enclosed by epoxy glue. Download Figure 1-1, TIF file.

10.1523/ENEURO.0394-22.2023.f2-1 Figure 2-1 Arduino User Interface. Our Arduino User Interface that allows adjustment of crucial ABS parameters. Oscillation length determines the alternation radius of the moving light. Frequency determines the speed of how quick the moving light alternates in a given oscillation length. RGB color mix allows adjustment not only of the color, but also of ABS brightness by determining RGB values between 0 and 255. Duration determines the overall duration of the preset ABS presentation after activation. LED strip selector allows the generation of different patterns that activate ABSs at different absolute and relative heights. Download Figure 2-1, TIF file.

10.1523/ENEURO.0394-22.2023.f2-2 Figure 2-2 Infrared videography enables semiautomated pixel change analysis during ABS stimulation. ***A***, Infrared (top) versus visible light (bottom) videographic images during ABS stimulation. Using infrared videography, ABS and associated light artifacts are invisible. Infrared floor illumination prevents shadowing and enhances the contrast between animals and the background. ***B***, Pixel change analysis on an example of parallel visible-light versus infrared videographic recordings shows massive artifacts during ABS stimulation. Inset, Without ABS stimulation, visible-light and infrared videography showed comparable detection of freezing (gray bars). ***C***, Moving shadows, fur reflexes, and ABS light movements themselves distort image analysis. ***D***, Semi-automatic freezing analysis of parallel recordings in visible versus infrared light during ABS stimulation [*n* = 12 (2 CSs per mouse, 6 mice in total)]. Freezing behavior shown in a slope graph with paired Hedge’s *g*. Download Figure 2-2, TIF file.

Movie 1. Different patterns of ABSs. The video shows 4 different 2MDR oscillation patterns that can be selected and adjusted to fit the experimental requirements. Horizontally, vertically, and double oscillatory or counter rotation patterns are shown. Note the variations of oscillation width, frequency, brightness, and color. 10.1523/ENEURO.0394-22.2023.video.1

The stimulus presentation is initiated by an electrical input signal that can be triggered via the GPIO pin to the microcontroller board (Uno, Arduino) either manually or electronically. Manual input trigger signals can be delivered by a simple push-button operation at the remote control (Figs. 1*E*, 2*B*, Extended Data Fig. 1-1*C*). Electronic input trigger signals (e.g., generated by TTL output boards of commercial behavioral setups) can be connected to the microcontroller board (Uno, Arduino) via BNC connection. This enables users to easily integrate the apparatus into existing rodent behavioral setups. We used our 2MDR system with a regular auditory fear conditioning (FC) system (Maze Engineers) and a TTL trigger unit (Maze Engineers). We isolated the fear conditioning box with acoustic foam (Flat Tec 2 cm, AixFoam) to optimize sound presentation. A custom-made infrared floor illumination panel based on 940 nm LEDs (Figs. 1*F*, 2*D*, Table 2) was placed under a semitransparent acrylic floor to increase the visual contrast of mouse behavior in video recordings and to facilitate behavioral analyses. We integrated two top-view cameras in the fear conditioning box above the conditioning chamber: an infrared camera (DMK camera, model 37BUX265, The Imaging Source; and lens model TPL 0620 6MP, The Imaging Source; 850 nm long-pass filter, model FEL0850, Thorlabs) recorded animal behavior. Freezing behavior was quantified in infrared videos based on detection of frame-to-frame pixel changes using ezTrack (Pennington et al., 2019). A second treatment monitoring camera (HD webcam model C310, Logitech) enabled the experimenter to visualize and manually center the position of ABSs in the head direction of mice during fear extinction (Movie 2). Dynamic 360° shifting is achieved by a rotary encoder that communicates with the microcontroller board (Uno, Arduino) via two GPIO pins (Fig. 2*B*). The fear conditioning chamber, camera recording, and 2MDR apparatus were synchronized in a master–slave architecture: the TTL output unit of the fear conditioning box generated TTL signals under the control of original software (Maze Origin, Maze Engineers). TTL signals were fed into a control console (Fiber Photometry Console, Doric) and visualized in its original software (Doric Neuroscience Studio). Consecutively, the control console triggered the infrared video recordings and synchronized presentations of conditioned stimuli (CSs) and ABSs. Before the start of ABS presentations, the experimenter chose, adjusted, and uploaded the suitable Arduino script on the microcontroller. TTL input to the microcontroller board (Uno, Arduino; https://www.arduino.cc) triggered the preset presentation of ABSs within the experiment.

**Table 2:** List of components necessary to build the infrared floor light

Item	Supplier/Product number	Quantity	Price (€)
Infrared LED	Conrad Electronic/EAN: 2050000038259	500	0.12 each
Europlatine breadboard	Conrad Electronic/EAN: 4016139261881	5	5.39
DC supply 24V 12W	Conrad Electronic/EAN: 4024559306952	1	8.39
Barrel jack adapter	Conrad Electronic/EAN: 4043619654215	1	2.24
Total price			97.58

Note: As a light floor, we used white acrylic glass (1 cm) with a light transmission of <30%.

Movie 2. Remote control of ABS rotation inside the treatment cylinder. The demonstrative video shows how the 2MDR remote control rotates preset ABSs inside the cylinder (first part) to center ABSs in head direction of a mouse that is turning quickly (second part) and that is freezing upon CS presentation (part 3). 10.1523/ENEURO.0394-22.2023.video.2

### Animals

In the study, 11- to 17-week-old male C57BL/6 wild-type mice were used, which were housed in small groups [maximum of four mice in one cage of an IVC (Individually Ventilated Cage) system] with food and water available *ad libitum* under a 12 h light/dark cycle (lights off at 7:00 A.M.). All experiments were conducted during the dark phase of the cycle. Two days before the start of the experiment, male mice were single housed to prevent that social transfer of fear between male mice may influence the individual ABS treatment responses (Smith et al., 2021). All animal procedures were approved by the local governing body (Regierungspräsidium Karlsruhe, Germany, Ref. 35-9185.81/G-83/21) and performed in accordance with the Heidelberg University animal care committee regulations. Efforts were made to minimize animal suffering and to reduce the number of animals used according to the “3Rs” principles. Experimenters were blinded to the identity of the mice during the analysis of behavioral tests.

### Behavioral experiments

We used our 2MDR system in combination with a fear conditioning setup during fear extinction experiments in mice (Curzon et al., 2009). Fear behavior was quantified as relative freezing time.

#### Habituation

Mice were transferred to a holding room neighboring the experimental room and rested for 1 h before and after experiments. For 3 consecutive days, mice were handled by the experimenter for a few minutes and habituated in context A (see subsection Experimental environments during fear conditioning) for 15 min/d. Before each experiment, mice were habituated for a short time (120 s) in the respective contexts.

Auditory FC (day 1) was performed in context A by pairing a CS (3 kHz tone, 30 s continuous, 75 dB) either (4 × 04 paradigm) four times with a soft unconditioned stimulus (US1; electric footshock, 0.4 mA, 1 s) or (5 × 07 paradigm) five times with a stronger electric footshock (US2; 0.7 mA, 1 s). CS and US coterminated, and CS–US combinations were presented at pseudorandomized intertrial intervals (ITIs) between 90 and 150 s. For experiments seen in Figures 4 and 5, we conditioned the mice using the 5 × 07 paradigm. To ensure comparable fear extinction behavior between different intervention groups, we excluded animals with a maximal relative freezing time <65% (∼1 SD below the overall mean relative freezing time) during the last three CS–US presentations at the FC end (5 of 56 animals in total).

#### Fear extinction

On day 2, we presented CS 20 times at pseudorandomized ITIs of 60–90 s and measured CS-mediated freezing behavior in context B (see subsection Experimental environments during fear conditioning). In CS control groups, the CS was presented without ABSs. In treatment groups, ABSs or extra-bright bilateral stimuli (XBSs) were paired with CSs from the second CS presentation onward. In contrast, we solely presented ABSs (19 times at pseudorandomized ITIs of 60–90 s) without CSs in mice of the ABS-only group on day 2. Without CS exposure in this experiment, we did not analyze CS-mediated freezing behavior in these mice on day 2.

#### Fear recall

On day 10, we presented 3 CS at pseudorandomized ITI of 90–150 s in context B and measured CS-mediated freezing behavior.

#### Fear renewal

At least 2 h after recall tests on day 10, we presented three CSs at pseudorandomized ITIs of 90–150 s in a novel context C (see subsection Experimental environments during fear conditioning).

#### Experimental environments during fear conditioning

To reduce the degree of contextual fear associations, behavioral experiments were performed in different contexts. Context A consists of a square experimental chamber (16 × 16.5 × 25 cm) with gray monochrome walls, a shock grid with metal rods (Maze Engineers), and white light illumination (30 lux). Context B is the round 2MDR cylinder (diameter, 20 cm; height, 20 cm) on a smooth acrylic glass base plate in no-visible-light conditions and scented with 1,4-cineol (Sigma-Aldrich). Context C consists of a square chamber (16 × 16.5 × 25 cm) with black and white striped walls (Maze Engineers), a textured, custom base floor plate, white light illumination (7 lux), and a rose blossom scent (scented creme, Rituals). All contexts were continuously illuminated by our infrared floor light panel.

### ABS treatment during fear extinction

The features of ABSs were preset in our custom-made Arduino script and uploaded to the microcontroller before the start of the experiment (Extended Data Fig. 2-1). We used the following settings for classic ABSs (see Fig. 4). In the Arduino script “Full_cylinder_horizontal_with_rotary_encoder,” we adjusted the oscillation length to nine LEDs used; the alternation frequency to 1 Hz; the stimulus color and brightness to red = 30, green = 30, and blue = 30; stimulus duration to 30 s; and activated LED strip 2. Extra-bright bilateral stimuli (see Fig. 5) consisted of increased channel values for red = 255, green = 255, and blue = 255. The duration of both CSs and ABSs was 30 s, and both ABSs and CSs started simultaneously. TTL signals automatically started ABS presentation that consisted of an oscillating light beam in which nine LEDs were switched on serially from left to right and vice versa with an alternation frequency of 1 Hz. The experimenter then manually presented these ABSs as central as possible in the head direction of the mice using the remote control.

### Freezing analysis

Infrared videography was recorded at a frame rate of 25 frames/s. Infrared video material enabled us to semiautomatically scored freezing behavior based on frame-to-frame pixel change with the open-source algorithm ezTrack (Pennington et al., 2019). The motion threshold and the freezing threshold of the animal were defined as described in the article by Pennington et al. (2019). We scored freezing if pixel changes (a correlate of animal movements) were below the freezing threshold for at least 25 consecutive frames (1 s). We analyzed the freezing time during CS presentation and scored relative freezing as a percentage of CS time [freezing (%) = freezing time (s)/30 s]. We initially validated whether infrared and visible light videography enables comparable freezing detection during the presentation ABSs (Extended Data Fig. 2-2). We simultaneously recorded animal behavior with our monitoring and infrared camera (Movie 3) during two CS-ABS presentations and performed semiautomated freezing analysis on both types of videographic recordings (Extended Data Fig. 2-2*A*,*B*). Infrared recordings facilitated the recording of freezing behavior throughout ABS stimulation, whereas visible light videography diminishes pixel change analysis during ABS treatments because of massive light artifacts [paired *t* test, *p* = 0, paired Hedges’ *g* = −2.78; *n* = 12 CSs (6 mice) each; Extended Data Fig. 2-2*D*]. In rare cases (three mice), we observed active escape behaviors in the 5 × 07 group in the first two FE sweeps, but not in other parts of the experimental approach.

Movie 3. Videographic recording during ABS treatment in visible light and IR light. The video shows a parallel recording of mouse behavior before and during CS presentation and ABS stimulation in visible light and infrared light. Furthermore, the video shows that our infrared floor light increases the contrast between animals and the background. 10.1523/ENEURO.0394-22.2023.video.3

### Statistics

The primary study aim was to test the hypothesis of whether classic ABS stimulation using 2MDR persistently reduces conditioned fear in mice. As a secondary exploratory study aim, we investigated the influence of physically altered XBS on long-term fear reduction. As a secondary subordinated aim, we also investigated whether ABS stimulation alone induces persistent fear reduction.

To account for the study structure and the control α, and to reduce the burden of animals, we analyzed the data using a hierarchical testing strategy with respect to our data seen in Figures 4 and 5 (Schulte Mönting, 2007). We ranked our null hypotheses in descending order of importance [(1) ABS effect; (2) XBS effect; (3) ABS-only effect) and sequentially tested these treatment hypotheses against the same CS control group. We only continued to test a less important null hypothesis if *p*_interaction_ < 0.05.

**Figure 3.**
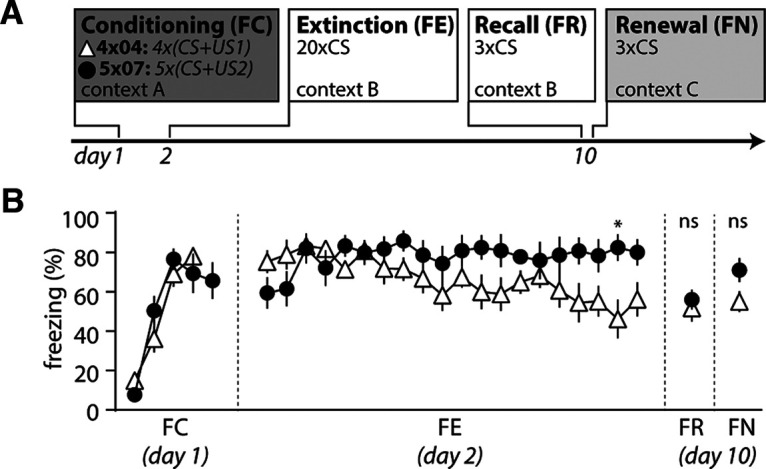
Establishment of a C57BL/6 mouse phenotype with impaired fear extinction. ***A***, Experimental timeline of fear conditioning, extinction, recall, and renewal. ***B***, Freezing behavior during FC, FE, FR, and FN in mice on fear conditioning with a 4 × 04 paradigm (white triangle; *n* = 9 mice) and a 5 × 07 paradigm (black circle; *n* = 9 mice). Mean ± SEM for every sweep. Asterisk indicates significant difference in two-way repeated measures with Sidak test.

**Figure 4.**
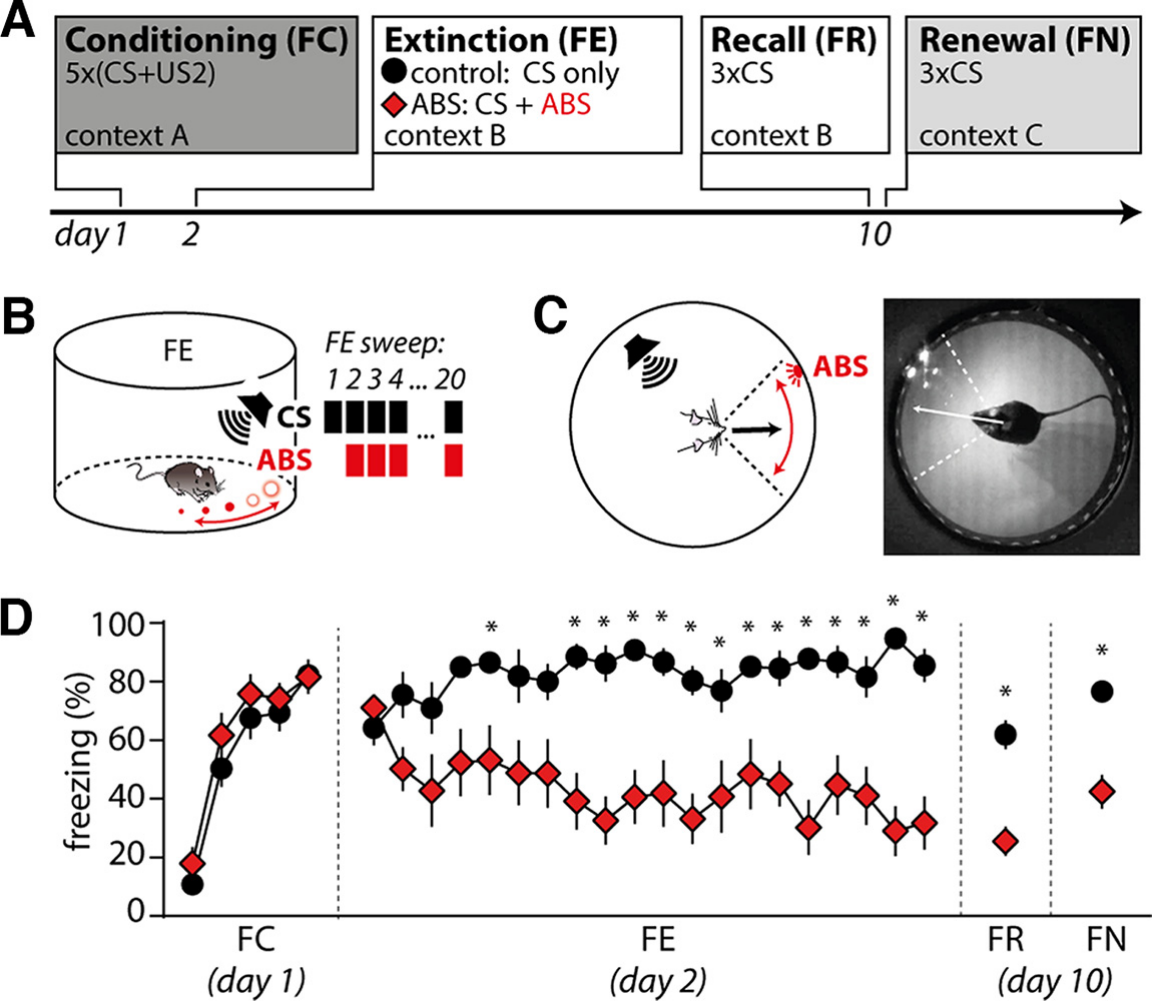
Classic ABSs persistently improve fear extinction in mice. ***A***, Experimental timeline. Mice of the ABS group were stimulated with classic ABSs (frequency, 1 Hz; light color, white) during CS presentation in fear extinction using our 2MDR system. Mice of the CS control group received 20 CS presentations only. ***B***, Schematic of fear extinction in the ABS group. ABSs started together with the second CS presentation. ***C***, Schematic and representative photograph showing how we centered ABSs in the head direction of mice during fear extinction in 2MDR cylinder. ***D***, Freezing behavior during FC, FE, FR, and FN in the CS control group (black circle, *n* = 10 mice) and the ABS group (red rhombus, *n* = 9 mice). Mean ± SEM for every sweep. Asterisks indicate significant group differences in a two-way repeated-measures ANOVA with Sidak test.

**Figure 5.**
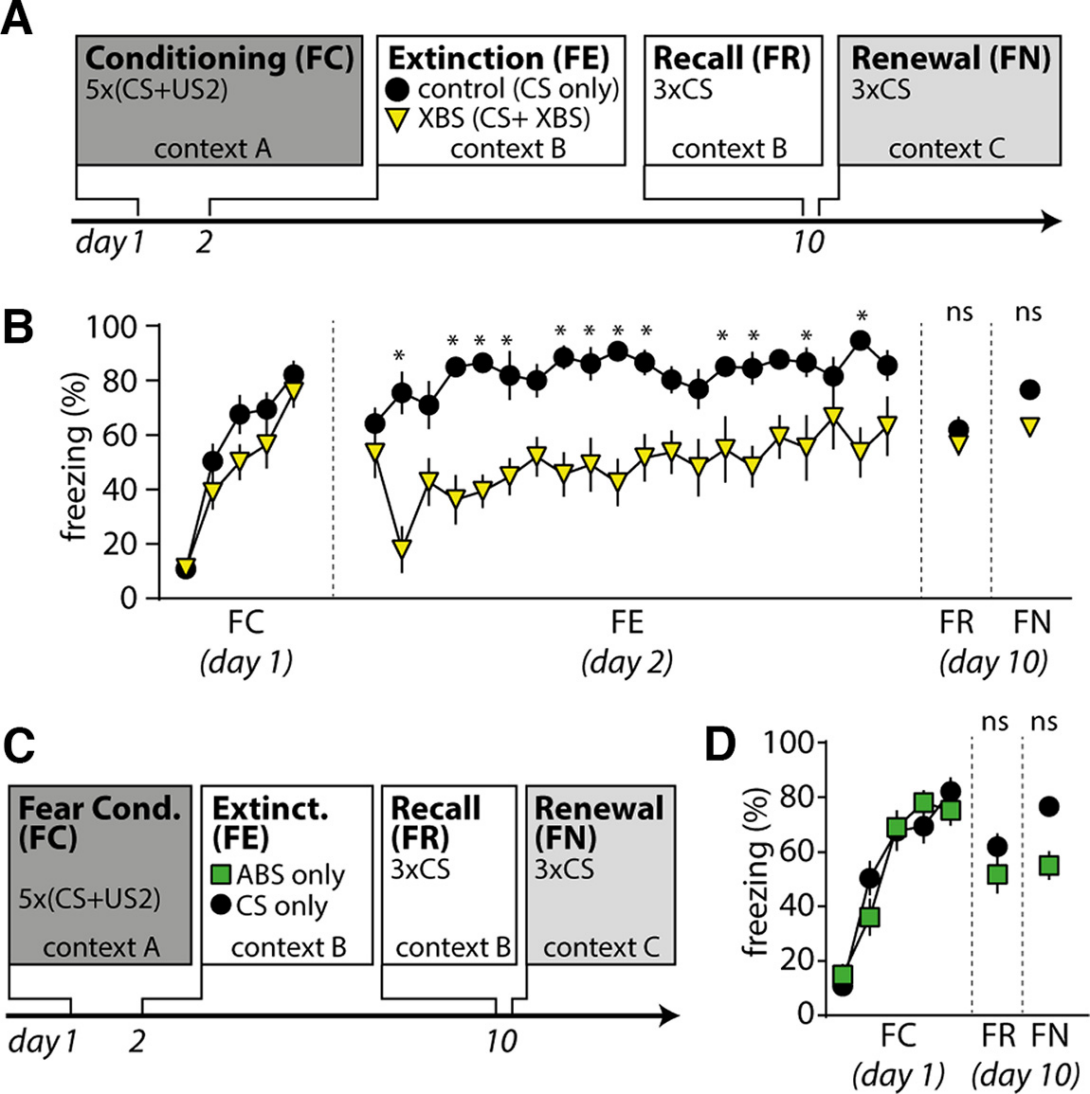
Both XBS treatment and ABS treatments independent from CSs do not elicit persistent anxiolytic effects in mice. ***A***, Experimental timeline. During fear extinction, mice of XBS group were stimulated with alternating extra-bright stimuli (XBS), and CS control mice received 20 CS presentations only. ***B***, Freezing behavior during FC, FE, FR, and FN in the XBS group (yellow triangle, *n* = 7 mice) and the same CS control mice (black circle, *n* = 10 mice). ***C***, During fear extinction, mice of the ABS-only group were stimulated with classic ABS (frequency, 1 Hz; light color, white) without CS presentation, the same CS control mice received 20 CS presentations only. ***D***, Freezing behavior during FC, FE, FR, and FN in the CS control group (black circle, *n* = 10 mice) and the ABS-only group (green square, *n* = 7 mice). Mean ± SEM for every sweep. Asterisks indicate significant group differences in a two-way repeated-measures ANOVA with Sidak test.

Statistical analyses were performed using GraphPad Prism 6 and www.estimationstats.com. Statistical significance was defined as a value of *p* < 0.05. Data are presented as the mean ± SEM if not indicated otherwise. Population size (*n*) is presented as the number of mice if not indicated otherwise. We tested our datasets for deviations from normality with the D’Agostino–Pearson test and Shapiro–Wilk test. As a statistical parameter for successful fear conditioning and the strength of US–CS association, we used the relative freezing time during the last FC sweep. The effects of each individual treatment (Figs. 3-5) were analyzed by a single two-way repeated-measures ANOVA followed by Sidak’s test for multiple comparisons and included the freezing data from the last FC sweep, FE, fear recall (FR), and fear renewal (FN).

Comparing the two groups (infrared vs visible light; Extended Data Fig. 2-2*D*), we computed the paired Hedges’ *g* values using www.estimationstats.com and displayed a slope graph and Gardner–Altman estimation plot. The respective mean differences were plotted on floating axes on the right as a bootstrap sampling distribution. The mean difference is depicted as a dot; the 95% confidence interval is indicated by the ends of the vertical error bar. Five thousand bootstrap samples were taken; the confidence interval is bias corrected and accelerated. We computed *p* values of two-sided permutation *t* tests. The *p* values reported are the likelihoods of observing the effect sizes, if the null hypothesis of zero difference is true. For each permutation *p* value, 5000 reshuffles of the control and test labels were performed.

### Data availability

All the files for building instructions, Arduino software, and bill of materials used in this study are available as Extended Data Figures 1-1, 2-1, and 2-2 and online (https://github.com/WielandLab).

## Results

### Establishment of a PTSD-like phenotype of impaired fear extinction in male C57BL/6 mice

Many studies showed impaired fear extinction in several mental disorders like anxiety disorders and PTSD (Shin and Liberzon, 2010). C57BL/6 mice express impairments of fear extinction depending on the amount of aversive experiences during fear conditioning (Myers and Davis, 2007). We thus set out to test the effect of two different fear conditioning paradigms on the freezing behavior during fear extinction to determine which one induces stronger impairment of fear extinction in male C57BL/6 mice (Fig. 3*A*). In fear conditioning experiments on day 1, male mice learned to associate a sound (CS) with a footshock (US) of two different intensities (4 × 04 paradigm: 0.4 mA, four pairings of US–CS; 5 × 07 paradigm: 0.7 mA, five pairings of US–CS). We conditioned either with the 4 × 04 paradigm (*n* = 9 mice) or the 5 × 07 paradigm (*n* = 9 mice) and then compared cue-associated freezing behavior during the FE test on day 2, and during FR and FN on day 10.

A two-way ANOVA revealed a significant interaction between the conditioning paradigm and freezing behavior, suggesting that fear conditioning with a 5 × 07 paradigm induces more freezing behavior, especially at the end of extinction experiments (two-way repeated-measures ANOVA with Sidak test; *F*_interaction(22,352)_ = 2.948; *p*_interaction_ < 0.0001; *n* = 9 mice each; Fig. 3*B*). Both fear conditioning paradigms induce comparable freezing behavior on day 10 when we presented CS both in a familiar context [Sidak test: *p*(FR) ≥ 0.9999; Fig. 3*B*] and in a novel context [Sidak test: *p*(FN) = 0.9425; Fig. 3*B*]. We conclude that male C57BL/6 mice express short-term, but not long-term, impairments in fear extinction depending on specific fear conditioning parameters. For the following experiments in Figures 4 and 5, we thus used the 5 × 07 fear conditioning paradigm.

### ABS treatments persistently improve fear extinction in mice

To validate the functionality of our 2MDR system and confirm the findings of Baek et al. (2019), we investigated whether repetitive pairing of classic ABSs with CSs during fear extinction can persistently improve fear extinction of mice (Fig. 4*A*,*B*). Classic ABSs consisted of bilateral visual stimuli (white light) that horizontally oscillated at a frequency of 1 Hz (Fig. 4*C*). After fear conditioning, ABS mice (*n* = 9 mice) showed similar freezing responses to untreated CS control mice (*n* = 10 mice; Fig. 4*D*). We then performed treatments with ABSs during fear extinction experiments (ABS group) and compared freezing behavior during extinction, recall, and renewal tests with CS control mice that did not receive treatment with ABSs. A two-way ANOVA revealed a significant interaction between the treatment and freezing behavior, suggesting that ABS-treated mice express significantly less freezing behavior during fear extinction compared with CS control mice (two-way repeated-measures ANOVA with Sidak test; *F*_interaction(22,374)_ = 2.792; *p*_interaction_ < 0.0001; CS control, *n* = 10; ABSs, *n* = 9; Fig. 4*D*). We conclude that classic ABS treatments quickly reduce cue-associated freezing behavior during fear extinction.

We then wanted to determine whether ABSs reduce freezing behavior based on distraction during CS presentation or persistently improve fear extinction in mice. During fear recall on day 10, ABS-treated mice expressed significantly less CS-associated freezing (in the absence of ABSs) in the known treatment context [Sidak test: *p*(FR) = 0.0186; Fig. 4*D*]. We conclude that stimulation with ABSs persistently improves fear extinction in the known treatment context.

We then investigated whether the persistent reduction of cue-associated freezing after ABS treatments depends on the contextual ABS treatment cues. During FN on day 10, ABS-treated mice expressed significantly less freezing upon CS presentation (in the absence of ABSs) even in a novel context [Sidak test: *p*(FN) = 0.0367; Fig. 4*D*]. We conclude that treatments with ABSs during fear extinction significantly and persistently improved extinction of cue-associated fear in mice.

### Increasing ABS brightness diminishes persistent anxiolytic effects of bilateral stimuli

We wanted to investigate whether physical stimulus properties such as stimulus brightness influence ABS-mediated anxiolytic effects. To generate extra-bright ABS (i.e., XBS), we thus increased stimulus brightness and kept all other properties of ABSs constant (Fig. 5*A*). Mice of the XBS group learned CS–US associations to a similar extent as CS control mice (CS control, *n* = 10; XBS, *n* = 7; Fig. 5*B*). We then performed XBS treatments during fear extinction experiments (XBS group) and compared freezing behavior during extinction, recall, and renewal tests with the same CS control mice that did not receive XBS treatment. A two-way ANOVA revealed a significant interaction between the treatment and freezing behavior, suggesting that XBS-treated mice express significantly less freezing behavior compared with CS control mice, especially at the beginning of fear extinction (two-way repeated-measures ANOVA with Sidak test: *F*_interaction(22,330)_ = 2.330; *p*_interaction_ = 0.0008; CS control, *n* = 10; XBS, *n* = 7; Fig. 5*B*). We then wanted to determine whether XBS treatments may persistently alter the extinction of conditioned fear in a known and an unknown context, similar to treatments with ABSs. At day 10, we recorded freezing behavior on CS presentation in the treatment context (FR) and in a novel context (FN), and showed that XBS treatment did not persistently alter freezing behavior on day 10 in both contexts [Sidak test: *p*(FR) > 0.9999; *p*(FN) = 0.9814; Fig. 5*B*]. We conclude that XBS stimulation induced short-lasting, but not long-lasting, fear reduction in mice.

### ABS treatments in the absence of CS exposure do not reduce conditioned fear

ABSs may directly induce long-lasting fear reduction independent of CS pairing. We thus performed ABS treatments without CS exposure (ABS-only group, *n* = 7 mice) in fear conditioned mice and compared freezing behavior during recall and renewal tests on day 10 with the same CS control mice that did not receive ABS-only treatment. A two-way ANOVA revealed no interaction between treatment and freezing behavior, suggesting that ABS treatments without CS exposure did not significantly alter long-term freezing responses in mice (two-way repeated-measures ANOVA with Sidak test; *F*_interaction(2,30)_ = 0.7450; *p*_interaction_ = 0.4833; Fig. 5*D*). We conclude that ABS treatments without simultaneous CS exposure do not directly reduce cue-associated fear in mice.

## Discussion

Here, we present 2MDR, a low-cost, open-source visual stimulation system for EMDR-like interventions in mice. 2MDR standardizes the application of multiple ABS variants and the behavioral assessment in rodent EMDR-like treatments. We provide detailed hardware-building instructions and open-source control software to ease the setup for inexperienced researchers. There is, to our knowledge, no commercial alternative to perform EMDR-like experiments in rodents.

Using 2MDR, we showed that classic ABSs persistently improved fear extinction in mice. These results not only proved the functionality of our device but also confirmed the groundbreaking findings of the study by Baek et al. (2019), as we showed comparable ABS-mediated anxiolytic effects. The present study primarily aimed to validate the custom-built behavioral setup, so only male mice were used as subjects. Although PTSD affects women twice as likely as men and sex differences are of considerable interest, they are outside the scope of the present article. A second set of experiments showed that anxiolytic effects depend on physical ABS properties as bright ABSs failed to persistently improve fear extinction. These different behavioral effects may be evoked by the activation of different visual circuits (Williams et al., 2021), different head/eye movements (Calancie et al., 2018; Holmes, 2019), or visual ABS features being inside/outside the mouse “alert range” (Yang et al., 2020). In conclusion, we show for the first time that the exact ABS composition is relevant for the behavioral effect of EDMR in mice, thereby providing a translational bridge to study the mechanisms of stimulus specificity (Matthijssen et al., 2019) and of anxiolytic effects of ABSs.

General principles of 2MDR are based on the first mouse model of EMDR-like ABS presentation (Baek et al., 2019). The researchers showed a setup that was designed to present classic ABSs to freely moving mice during fear extinction. The setup in the study by Baek et al. (2019) consisted of a black treatment cylinder of 20 cm diameter inside a fear conditioning chamber in which lights are switched off.

Despite many similarities between both animal models, 2MDR offers some key advantages over the model of Baek et al. (2019). First, Baek et al. (2019) used only a single row of white LEDs at a fixed distance above the ground to present alternating or flickering visual stimuli. A key advantage of 2MDR is the use of programmable, multirow RGB LED strip arrays that enable the testing of more visual stimulus characteristics such as stimulus width, color, brightness, pattern, duration, speed, and direction of stimulus movement and oscillation. In particular, multirow arrays can present ABSs in vertical or angled directions, at different heights and in counter-rotation. Although an assessment of treatments with different ABS variants is of considerable interest, it is outside the scope of the present article.

Baek et al. (2019) showed that ABSs activate SC neurons. Yet, different SC circuits are activated depending on visual stimulus characteristics (e.g., the position within the visual field) motion direction and velocity, stimulus color, and size (Dräger and Hubel, 1975; Wang et al., 2015; Ito et al., 2017; Cang et al., 2018; Choi and Priebe, 2020; Doykos et al., 2020; Lee et al., 2020; Li et al., 2020; Yang et al., 2020; Williams et al., 2021). As SC outputs and inputs are topographically mapped (Ito et al., 2017; Li et al., 2020; Benavidez et al., 2021), it is possible that ABS variants activate specific neuronal populations with different potency.

Second, Baek et al. (2019) used an approach in which the experimenter chose between one of four quadrants in which ABSs were presented. ABS presentation could not continuously be centered to the head direction of mice. In contrast, we use a remote control to smoothly rotate ABSs in 360° inside the treatment cylinder. This rotary control can dynamically center ABSs in head direction of freely moving mice, thereby creating a more standardized situation similar to the human EMDR treatment situation. Recent studies show that mice have a sophisticated control of their visual field during freely moving behavior (Wallace et al., 2013; Meyer et al., 2018, 2020; Michaiel et al., 2020; Holmgren et al., 2021). During attack, head movements initiate most eye movements pursuing prey (Michaiel et al., 2020). Yet, mice can also express directed saccades to a unilateral stimulus (Zahler et al., 2021). Eye movements center the cue in a small functional focus area within the visual field (Holmgren et al., 2021). Yet, it is unknown whether mice show pursuit saccades on ABS presentation (Holmes, 2019). With respect to a highly differentiated oculomotor system in mice, it seems necessary to increase the precision of ABS presentation for future studies.

Third, Baek et al. (2019) recorded animal behavior in visible light. Animal videography thus contained ABS light signals that induce strong videographic artifacts. These artifacts bias an automated pixel change analysis toward an overestimation of ABS treatment effects as freezing during ABS treatment is undetectable. Baek et al. (2019) solved this issue by manually scoring freezing behavior during ABS treatments, yet this manual scoring (especially when ABSs are visible in the video) may have big disadvantages in terms of blinded behavioral analysis. In contrast, our 2MDR system uses infrared videography in combination with a custom-made infrared floor illumination to record and semiautomatically analyze freezing behavior of mice throughout the experiments.

However, it should be noted that 2MDR uses LED strips for light generation. Although they offer advantages in terms of durability, easy setup, building costs, and adaptability, they are restricted to a certain number of pixels per row. Other solutions like flexible active-matrix organic LED displays may be favorable to generate detailed ABSs in high resolution. Yet, to better understand the principles of how ABS properties interact with the brain, we prioritized LED strips over high-resolution displays.

It is important to highlight the versatility of our visual stimulation system for future experiments, and their standardization as 2MDR size can easily be scaled up for experiments in larger rodents like rats. 2MDR has a key value to better understand the physiology of how ABSs interact with the brain, whether different neural circuits can be stimulated with certain ABS variants and whether ABS variants may differentially improve motivational, nociceptive, or social behaviors. Together, 2MDR can facilitate EMDR-like interventions and future studies exploring whether visual stimuli can be used as a differential, noninvasive brain stimulation method in rodents.

## Synthesis

Reviewing Editor: Jonathan Lee, University of Birmingham Decisions are customarily a result of the Reviewing Editor and the peer reviewers coming together and discussing their recommendations until a consensus is reached. When revisions are invited, a fact-based synthesis statement explaining their decision and outlining what is needed to prepare a revision will be listed below. The following reviewer(s) agreed to reveal their identity: Carl Stevenson.

The authors have addressed many of the comments on the original submission but others haven’t been addressed sufficiently and we now have other concerns about the data re-analysis in the revision.

Major:

The data analysis now includes the last CS-US trial during conditioning, the extinction trials, and the fear recall and renewal tests altogether in the same analysis. In our view it would be more appropriate to have separate analyses for the conditioning data (for Fig 4-5, this isn’t needed for weak vs strong conditioning in Fig 3), the extinction data, and the recall and renewal data. Analysis of the full conditioning datasets (Fig 4-5 only) would be useful to show no group differences in freezing before extinction. Analyzing the recall and renewal datasets together, but separately from the rest of the data, will also increase the likelihood of pulling out significant group differences (e.g. possibly Fig 3B and 5B but especially for Fig 5D) and is perfectly defensible as an analysis strategy.

An ABS only control has now been included (Fig 5D) but the data from the same CS only control group still seems to be used in the separate analyses on the effects of ABS + CS (Fig 4B), XBS + CS (Fig 5B), and ABS only (Fig 5D). As outlined in the comments on the original submission, this is inappropriate. Ideally data from separate control groups should be used for each comparison, or else the data can be combined in the same analyses. For example, the extinction data from the CS only control, ABS + CS, and XBS + CS groups can be combined together in the same analysis, with this likely still showing higher freezing in the CS only controls vs the ABS + CS and XBS + CS groups. Similarly, the recall and renewal data from the CS only control, ABS only control, ABS + CS, and XBS + CS groups can be combined together in the same analysis. If doing this then the fear conditioning data for all of the groups can also be combined in the new figure and statistical analysis. This would also be much more straightforward than the ‘hierarchical testing strategy’ described as justification for using the data from the same control group in each different analysis.

Minor:

There seems to be inconsistency between the Methods and Results / Table 1 on the numbers of animals used in the first experiment comparing the effects of weak vs strong conditioning. In the Methods it states that n=30 animals were used, with n=5 omitted from the analysis due to low freezing during fear conditioning. But in the Results / Table 1 it states that n=9/group, so n=18 in total, were used. This discrepancy needs to be clarified.

‘Control group’ throughout refers to the extinction controls (i.e. CS only during extinction) but this is potentially confusing since ABS controls (i.e. ABS only during extinction) are now also included. I suggest that ‘control group’ is renamed to CS controls or similar to make this clearer.

In the Methods, line 243, the two groups referred to should be included for clarity.

Table 1 is now clearer but it’s debatable if it’s actually needed along with the relevant text on the statistical analysis included in the Methods, Results, and Figures. It can be omitted in whole or in part (i.e. omit post-hoc test results).
