# Supplementary figures and images for: 2MDR, a Microcomputer-Controlled Visual Stimulation Device for Psychotherapy-Like Treatments of Mice
Source: eNeuro. 2023 Jun 2;10(6):ENEURO.0394-22.2023. doi: 10.1523/ENEURO.0394-22.2023 (PMC10246761; doi:10.1523/ENEURO.0394-22.2023)

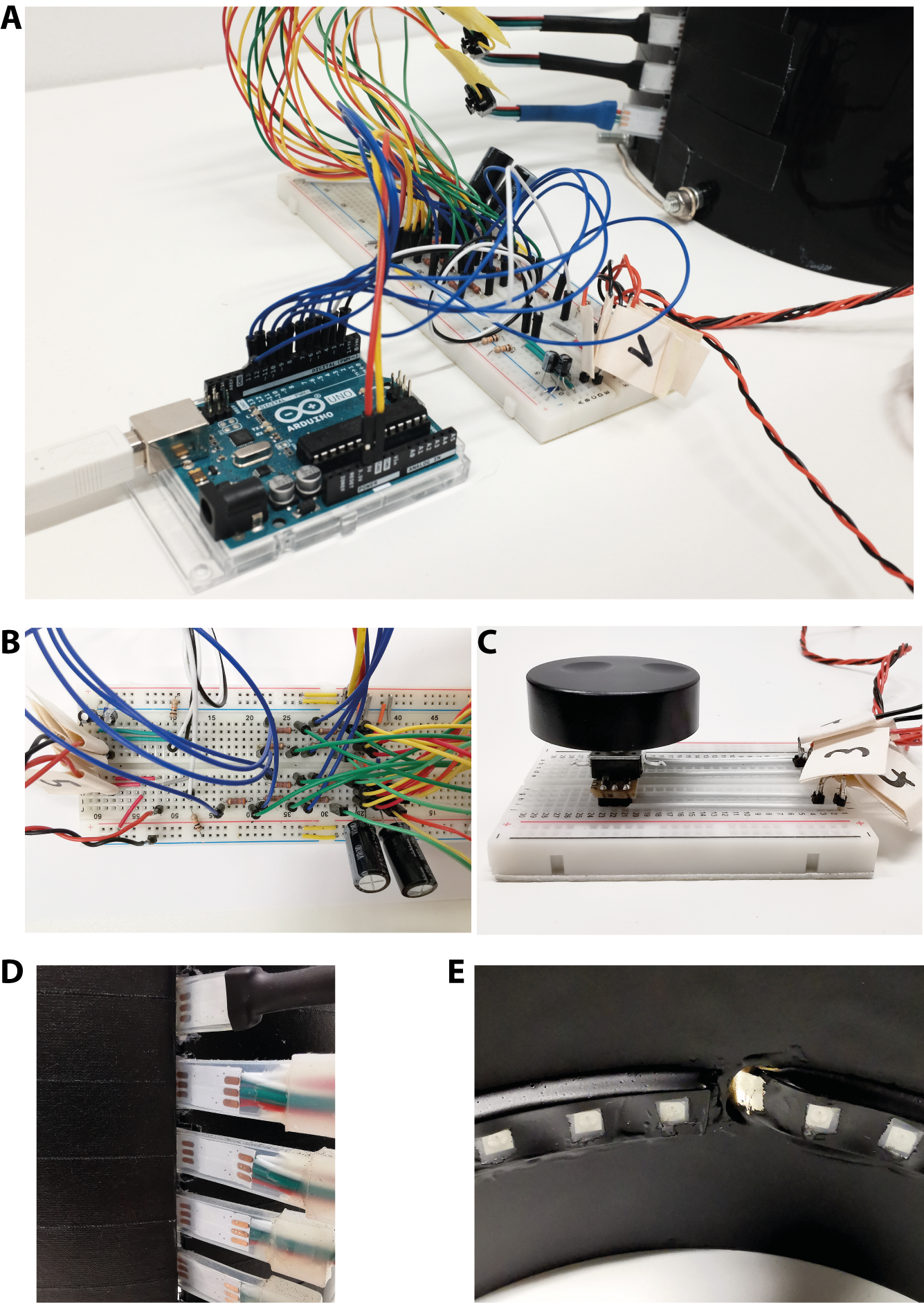

Supplement: Figure 1-1 — 2MDR hardware. Photographs showing hardware details of 2MDR. A, Details of microcontroller und breadboard. B, Breadboard shown in high resolution. C, Remote control–rotary encoder connections. D, E, Slit-like (D) and hole-like (E) entry of LED strip to cylinder. The hole-like entry was enclosed by epoxy glue. Download Figure 1-1, TIF file. [file enu-eN-OTM-0394-22-s07.tif]

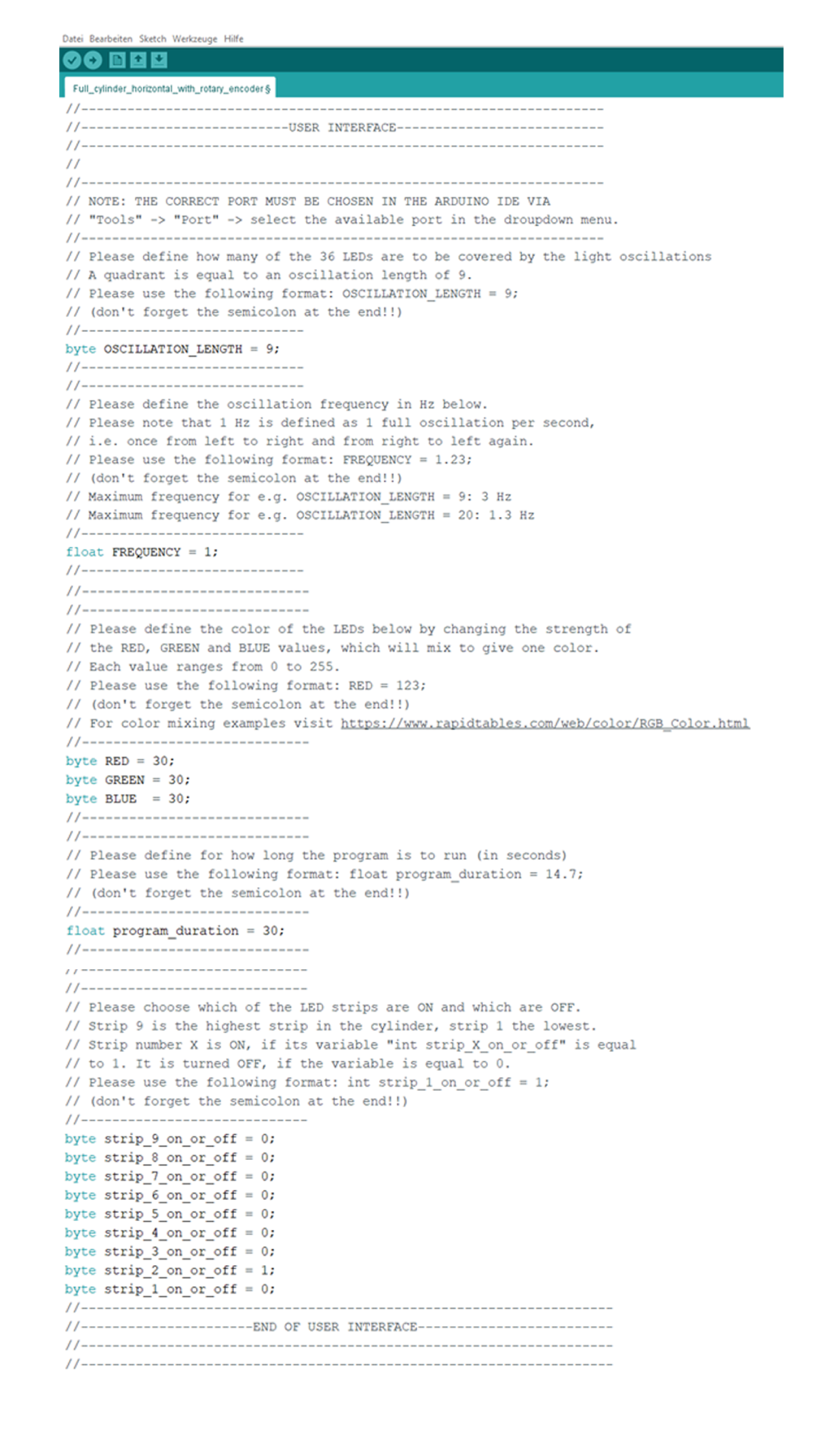

Supplement: Figure 2-1 — Arduino User Interface. Our Arduino User Interface that allows adjustment of crucial ABS parameters. Oscillation length determines the alternation radius of the moving light. Frequency determines the speed of how quick the moving light alternates in a given oscillation length. RGB color mix allows adjustment not only of the color, but also of ABS brightness by determining RGB values between 0 and 255. Duration determines the overall duration of the preset ABS presentation after activation. LED strip selector allows the generation of different patterns that activate ABSs at different absolute and relative heights. Download Figure 2-1, TIF file. [file enu-eN-OTM-0394-22-s06.tif]

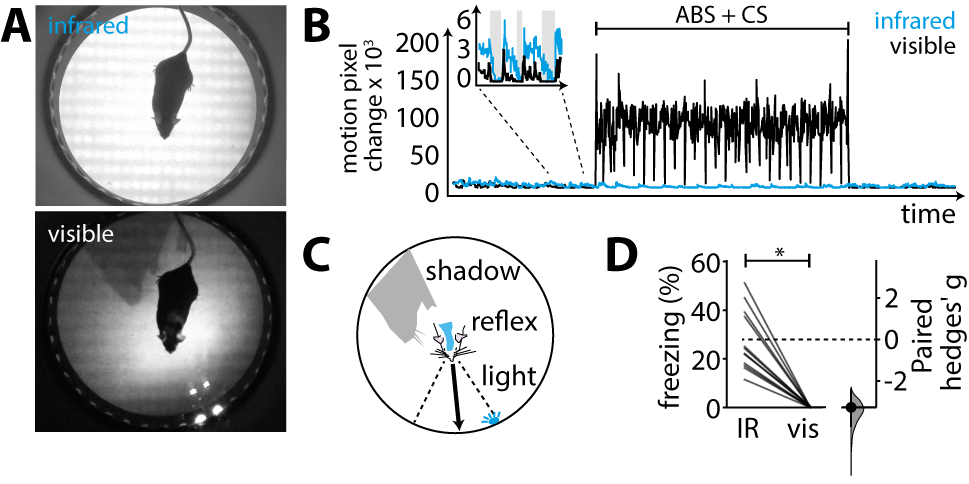

Supplement: Figure 2-2 — Infrared videography enables semiautomated pixel change analysis during ABS stimulation. A, Infrared (top) versus visible light (bottom) videographic images during ABS stimulation. Using infrared videography, ABS and associated light artifacts are invisible. Infrared floor illumination prevents shadowing and enhances the contrast between animals and the background. B, Pixel change analysis on an example of parallel visible-light versus infrared videographic recordings shows massive artifacts during ABS stimulation. Inset, Without ABS stimulation, visible-light and infrared videography showed comparable detection of freezing (gray bars). C, Moving shadows, fur reflexes, and ABS light movements themselves distort image analysis. D, Semi-automatic freezing analysis of parallel recordings in visible versus infrared light during ABS stimulation [n = 12 (2 CSs per mouse, 6 mice in total)]. Freezing behavior shown in a slope graph with paired Hedge’s g. Download Figure 2-2, TIF file. [file enu-eN-OTM-0394-22-s05.tif]
